# Face recognition’s practical relevance: Social bonds, not social butterflies

**DOI:** 10.1016/j.cognition.2024.105816

**Published:** 2024-06-21

**Authors:** Laura M. Engfors, Jeremy Wilmer, Romina Palermo, Gilles E. Gignac, Laura T. Germine, Linda Jeffery

**Affiliations:** a Justice and Society, University of South Australia, Adelaide, SA, Australia; b School of Psychological Science, University of Western Australia, Perth, WA, Australia; c Department of Psychology, Wellesley College, Wellesley, MA, USA; d Institute for Technology in Psychiatry, McLean Hospital, Belmont, MA, USA; e Department of Psychiatry, Harvard Medical School, Boston, MA, USA

**Keywords:** Extraversion, Social network size, Social network quality, Face identity recognition memory ability

## Abstract

Research on individual differences in face recognition has provided important foundational insights: their broad range, cognitive specificity, strong heritability, and resilience to change. Elusive, however, has been the key issue of practical relevance: do these individual differences correlate with aspects of life that go beyond the recognition of faces, per se? Though often assumed, especially in social realms, such correlates remain largely theoretical, without empirical support. Here, we investigate an array of potential social correlates of face recognition. We establish social relationship quality as a reproducible correlate. This link generalises across face recognition tasks and across independent samples. In contrast, we detect no robust association with the sheer quantity of social connections, whether measured directly via number of social contacts or indirectly via extraversion-related personality indices. These findings document the existence of a key social correlate of face recognition and provide some of the first evidence to support its practical relevance. At the same time, they challenge the naive assumption that face recognition relates equally to all social outcomes. In contrast, they suggest a focused link of face recognition to the quality, not quantity, of one’s social connections.

## 1. General introduction

The popularity of human face recognition as a research topic stems largely from its perceived importance to everyday life, particularly in social realms. It is easy to imagine that rapid, effortless recognition of faces could be a social asset—for example, by helping a person to make or maintain social connections. Yet is excellent face recognition really as important as it seems? Humans are remarkably creative and resilient; perhaps there exist other, equally effective routes to person recognition and/or social connection. Given the degree to which face recognition research is motivated by the hypothesis of social importance, a direct empirical test of that hypothesis seems warranted. Such a test is the first aim of the present work.

Beyond the question of *whether* face recognition is socially important is the question of *how* it may be socially important? Does it, for example, support the socially extraverted personality of the “social butterfly”? Does it expand the sheer number of social connections one is capable of maintaining? Does it help one to build an “inner circle” of strong social relationships to turn to in times of need? The second aim of the present work is to test the three specific mechanistic hypotheses suggested by the latter questions: that face recognition impacts (a) extraversion, (b) social network quantity (defined as the sheer number of persons interacted with at least bimonthly), and/or (c) social network quality (defined as the number of persons one could turn to for material or social support during adversity).

We take an individual differences approach to test these hypotheses (Wilmer, 2008). We start from the premise that if face recognition impacts an aspect of social life, then individual differences in face recognition, which are known to be large (Wilmer, 2017), should correlate with that aspect of social life. Since such an individual differences approach is correlational, it will not prove a particular causal direction. For example, it will not prove that face recognition impacts social life, rather than the other way around. Yet correlation is a precondition for causation; it reveals whether, where, and to what degree, causation could potentially exist. The sizes of correlations therefore define the magnitude of potential impact. Additionally, because sustained experimental manipulations of a person’s face recognition ability are probably neither feasible nor ethical, correlational studies may be the best available source of evidence for an impact of face recognition on social life.

How subtle should we expect individual differences based correlations to be? A systematic analysis of 780 articles on individual differences in personality and social psychology suggested benchmark correlations of 0.10 (“small”), 0.20 (“medium”), and 0.30 (“large”), due to their proximity to the 25th, 50th, and 75th percentile correlation values (Gignac & Szodorai, 2016). With these benchmarks in mind, we recruited samples sufficiently large to estimate results with reasonable precision, and we also replicated key results.

Efforts to establish a link between face recognition ability and social behaviour hold significant potential benefits. Social relationships are not only an important life outcome in themselves (Jenkins, Jono, Stanton, & Stroup-Benham, 1990); they also predict future success (Collins & van Dulmen, 2006; Masten, Desjardins, McCormick, Kuo, & Long, 2010), mental and physical health (American Psychiatric Association, 2013; Cohen, 1988; Holt-Lunstad & Smith, 2012; Miller, Chen, & Cole, 2009; Umberson & Karas Montez, 2010), happiness (Fowler, Christakis, Steptoe, & Diez Roux, 2009) and life satisfaction (Calmeiro, Camacho, & de Matos, 2018; Gadermann et al., 2016). Further, social skills and social experience, broadly defined, impact decision-making in numerous consequential contexts, including education (Fang, Sun, & Yuen, 2016; Langenkamp, 2010), healthcare (Freshman & Rubino, 2004; Mundt & Zakletskaia, 2014), and hiring (Calvo-Armengol ´ & Jackson, 2004; Castilla, 2005). Given the importance of social connections, it is of significant interest to ask whether and how they may relate to face recognition.

What existing sources of evidence motivate a theory that face recognition could impact social experience? One such source is comparative research, which shows that face recognition increases with social complexity. Rhesus monkeys, for example, show limited individual recognition skills, likely sufficient for their stable hierarchical structures (Parr, 2011; Rossion & Taubert, 2019). Chimpanzees, in contrast, have both more expert face recognition and larger, more fluid social networks (Parr, 2011). Humans exhibit still further complexity, in both social connections and face recognition architecture (Rossion & Taubert, 2019). A second source of evidence is brain research. Activity in core face recognition regions have been repeatedly linked with estimates of an individual’s social network size: (1) The fusiform face area, an area critical for holistic, specialised, facial encoding (Haxby, Hoffman, & Gobbini, 2000; Rapcsak, 2019; Rossion, Jacques, & Jonas, 2023), demonstrates greater activation and connectivity (Bickart, Hollenbeck, Barrett, Dickerson, & B. C., 2012) as well as increased gray matter volume (Kwak, Joo, Youm, & Chey, 2018) in people with more social contacts. (2) The anterior temporal lobe regions, involved in identity-specific knowledge retrieval (Olson, McCoy, Klobusicky, & Ross, 2013), correlates with individual differences in the number of social contacts (Kanai, Bahrami, Roylance, & Rees, 2012; Lewis, Rezaie, Brown, Roberts, & Dunbar, 2011). (3) The orbitofrontal cortex, involved in evaluating the personal reward value and relevance of faces (Rapcsak, 2019; Wang et al., 2021) demonstrates correlations with having a greater number of supportive social relationships (Hampton, Unger, Von Der Heide, & Olson, 2016; Liu et al., 2018; Von Der Heide, Vyas, & Olson, 2014). (4) The amygdala, involved in extracting social and emotional significance from faces (Wang et al., 2016; Wang, Zhu, Song, & Liu, 2017), and the ventro-medial prefrontal cortex, involved in top down monitoring over the operations of the temporal lobe face perception and memory networks (Rapcsak, 2019), have been implicated in individual differences in real-world social networks (Bickart, Wright, Dautoff, Dickerson, & Barrett, 2011; Kanai et al., 2012; Lewis et al., 2011; Liu et al., 2018; Noonan, Mars, Sallet, Dunbar, & Fellows, 2018; Von Der Heide et al., 2014; Wang et al., 2021). Taken together, these results from comparative and brain research suggest a possible link between face recognition and social connections. Yet they do not directly demonstrate such a link.

Three lines of evidence exist that more directly associate face recognition with social connections. First, people with developmental prosopagnosia (DP) have significant social challenges, including avoidance of social situations, difficulties in interpersonal relationships, and restricted social circles (Dalrymple et al., 2014; Diaz, 2008; Fine, 2012; Yardley, McDermott, Pisarski, Duchaine, & Nakayama, 2008). Second, high-quality, individuating interracial experiences, such as those gained through numerous interracial friendships, are associated with improved recognition of other-race faces (Bukach, Cottle, Ubiwa, & Miller, 2012; Hancock & Rhodes, 2008; Walker & Hewstone, 2006; Walker, Silvert, Hewstone, & Nobre, 2008) whereas passive exposure to other racial groups does not (Brunet, Taddei, Py, Paubel, & Tredoux, 2022; Ng & Lindsay, 1994). Third, autistic individuals, who show a variety of social deficits, show selective impairments in face recognition (i.e., relative to their non-face recognition ability, Griffin, Bauer, & Scherf, 2021; Tang et al., 2015; Weigelt, Koldewyn, & Kanwisher, 2012). These three lines of evidence, while suggestive, may have limited generalizability to everyday face recognition. They each focus on select populations with complex, potentially multifaceted disorders (such as Autism and DP), or perception of stimulus classes (such as infrequently encountered races or ages) that do not explicitly reflect a person’s common, everyday experience. As a result, while these studies provide potential insights into the effects of restricted experience on face recognition ability, they do not directly inform us about how natural variation in face recognition ability relates to real-world social functioning in daily life (e.g. relationships, interactions). Thus, the everyday functional correlates of face recognition ability remain poorly understood.

To address this gap, the present work had two complementary aims. First, we sought to determine whether face recognition ability relates to the quantity or quality of one’s social network. This was done in Studies 1 and 2. We assessed social networks via three well-established measures commonly used in the brain imaging studies mentioned above, that linked variations in social connections to face recognition-related brain regions (Bickart et al., 2011; Bickart et al., 2012; Hampton et al., 2016; Liu et al., 2018; Von Der Heide et al., 2014). The first measure was the Social Network Index (SNI, Bickart et al., 2011), which records the number of friends and acquaintances with whom one regularly interacts (often referred to as the ‘sympathy group’, Dunbar & Spoors, 1995). The second measure was number of Facebook friends.^1^ Previous work has indicated that online social network size relates to offline social interactions (Dunbar, 2016). We consider SNI and Facebook friends to measure the *quantity* of one’s social connections. The third measure was the Norbeck Social Support Questionnaire (NSSQ, Norbeck, Lindsey, & Carrieri, 1981), which estimates the number of intimate relationships who would readily provide support (often referred to as the ‘support group’, Dunbar & Spoors, 1995). We consider NSSQ to measure the *quality* of one’s social connections.

Our second complementary aim was to investigate the relationship between extraversion - a common personality trait measure of social behaviour - and face recognition. This was done in Studies 2–4. Recognising that previous research has offered mixed findings on the link between unfamiliar face recognition and extraversion, we began with a meta-analysis of these five studies (reported in Table 1) to provide clarity. We found a small, positive correlation, with a sample-size-weighted mean of *r* = 0.11 (total *N* = 1481, 95% CI [0.06,0.16]).^2^ However, a recent review found that mean meta-analytic effect sizes in studies of human behaviour are nearly three times inflated compared to large-scale replication studies (Kvarven, Strømland, & Johannesson, 2019). To critically examine the robustness of extraversion as a social correlate of face recognition ability, we therefore conducted three methodologically diverse replication studies involving multiple measures (Studies 2–4) and one very large sample (Study 4). Within this aim, Study 2 assessed the association between extraversion and both social network quantity and quality. Quantity was operationalised as the number of regular contacts assessed by the SNI (Bickart et al., 2011), Dunbar’s Number (Dunbar, 1993) and number of Facebook friends. Given extraverts’ tendency to be more outgoing and participative in social activities (Argyle & Lu, 1990), and based on previous work showing extraversion relates positively to regular contacts (Molho, Roberts, de Vries, & Pollet, 2016), we hypothesised a positive association between extraversion and these quantity measures. However, we did not necessarily predict a similar link with quality, operationalised as the number of supportive, emotionally close ties estimated with the NSSQ (Norbeck et al., 1981). This was based on prior findings that extraversion is not significantly associated with the number of support group members (Molho et al., 2016). Study 3 then aimed to identify social personality traits related to face recognition ability using a broad battery of sociability measures. Finally, Study 4 examined the correlation between face recognition ability and extraversion, leveraging a web-recruited sample that was age-diverse and substantially larger than the samples used in previous literature (*N* = 2028). These steps were taken to strengthen the generalisability and reliability of our findings.

Together, these studies provide a thorough examination of the long-hypothesised link between individual differences in face recognition ability and aspects of everyday social functioning. By leveraging multiple large samples from the general population and multiple complementary measures assessing both unfamiliar and familiar face recognition performance as well as diverse indices of social functioning, we sought evidence of connections between face recognition abilities and indicators of social functioning that have remained difficult to reliably establish. Mapping out these relationships allows for a fuller understanding of how face recognition skills may scaffold everyday interpersonal interactions essential for navigating social environments.

## 2. Studies 1 and 2

In Studies 1 and 2, we investigated the relationship between unfamiliar and familiar face recognition abilities and the quantity and quality of one’s social experience. Unfamiliar face recognition ability refers to the ability to learn and subsequently recognise a previously unfamiliar face in an experimental setting (Johnston & Edmonds, 2009). To measure this, we used the Cambridge Face Memory Test (CFMT), a renowned and psychometrically sound measure in the field (Duchaine & Nakayama, 2006). The CFMT is highly regarded for its reliability and validity, making it a well-established choice for assessing this specific aspect of face recognition (Wilmer et al., 2012). Conversely, familiar face recognition ability involves a more extensive learning phase and encompasses the ability to recognise faces encountered in everyday environments and across various occasions and contexts, such as friends, family members, and celebrities (Johnston & Edmonds, 2009). To assess this, we used a famous faces recognition task designed for the participant sample.

To assess participants’ social functioning across both studies, we used four well-validated, widely used measures chosen for their extensive use in the social neuroscience literature linking real-world social networks with face recognition regions. The SNI (Bickart et al., 2011; Bickart et al., 2012), Facebook friends (Kanai et al., 2012; Von Der Heide et al., 2014), NSSQ (Hampton et al., 2016; Von Der Heide et al., 2014), and a free recall Dunbar’s Number measure (Lewis et al., 2011; Von Der Heide et al., 2014). The SNI (Cohen, 1997) gauged regular contacts interacted with at least once every 2 weeks, Facebook friends assessed number of online connections, the NSSQ (Norbeck et al., 1981) estimated supportive inner circle relationships, and Dunbar’s Number (Dunbar, 1993) assessed the number of individuals a person can maintain in their social circle. Our first hypothesis for both studies was that if face recognition ability is related to the quantity of social interaction, there would be a statistically significant association between the size of an individual’s social network (both in real-life and online) and unfamiliar and familiar face recognition performance. Our second hypothesis was that if face recognition ability relates to having more intimate, supportive social connections, individuals reporting a greater number of emotionally close relationships assessed by the NSSQ would demonstrate better unfamiliar and familiar face recognition performance.

Our methodology across the four studies addresses several important factors. Firstly, we acknowledge the influence of age on both face recognition ability (Germine, Duchaine, & Nakayama, 2011) and our social factors (McCrae et al., 1999; Roberts, Wilson, Fedurek, & Dunbar, 2008), by controlling for age across all studies. We achieved this by regressing age out of all variables using a first order (Studies 1–3) and second order (Study 4) regression. This allowed us to examine the association between social experience and face recognition ability irrespective of age (Halberda, Ly, Wilmer, Naiman, & Germine, 2012). We also focused on recruiting participants aged 18–33 years old in Studies 1–3, as face recognition ability typically starts to decline around the age of 33 (Germine et al., 2011). Second, we aimed to recruit a balanced sample of males and females. Nevertheless, there was an overrepresentation of females in Study 1 (61 out of 93 participants), Study 3 (122 females out of 203 participants), and Study 4 (1443 females out of 2028 participants). Only Study 2 had a more equal gender distribution (47 males out of 101 participants). Finally, we took steps to minimise potential confounding effects related to the race of faces used in our face recognition ability measures. To achieve this, we specifically recruited participants from predominantly Caucasian countries or those with predominantly Caucasian heritage. This approach aimed to mitigate the impact of unfamiliarity and limited exposure to faces of different races on the results of our face recognition ability measures.

### 2.1. Study 1

#### 2.1.1. Study 1 method

##### 2.1.1.1. Participants.

Ninety-five participants between the ages of 18–33 years were recruited from the University of Western Australia community. All participants reported normal or corrected to normal vision. Two participants declined to complete the NSSQ, resulting in their exclusion from data analyses. The final sample consisted of 93 participants (32 males, age range 18–32 years, M_age_ = 20.40 yrs., SD_age_ = 1.98).

##### 2.1.1.2. Measures

###### 2.1.1.2.1. Face recognition ability measures

####### 2.1.1.2.1.1. Unfamiliar face recognition ability

We used the Cambridge Face Memory Test (CFMT; Duchaine & Nakayama, 2006). In the CFMT, participants study and then attempt to recognise six Caucasian male target faces in a 3-alternative forced-choice format. The first stage tests participants with probe faces that are an identical image to the target face. The second stage contains probe faces that are shown from different viewpoints and under different lighting conditions. The third and most difficult stage contains probe faces with the addition of visual noise. It is a widely used, well-validated, reliable measure of face recognition ability that is well suited for capturing individual differences in face recognition (Wilmer et al., 2012). Reliability for this sample was excellent: inter-item α = 0.88.

####### 2.1.1.2.1.2. Familiar (famous) face recognition ability

We used a famous face recognition test to assess familiar face recognition ability (Engfors & Churches, 2012). This task is a paper-and-pencil formatted measure of 30 famous faces (15 male, 15 female) that an Australian adult sample would have had some exposure to via magazines, television, and film. All faces were edited to gray-scale, and all hair was digitally removed, so only the faces were shown. Participants were instructed to identify each face by providing a name or some other uniquely identifying information. After the test, participants were informed of the identity of the faces (names and identifying information for each identity were presented along with the image of the face presented in color, with hair) and were required to indicate whether they were familiar or not. Accuracy scores were then calculated as a percentage of faces recognised from faces known. The task is suitably difficult for a non-clinical sample, and demonstrates wide variability (*M* = 54.4%, *SD* = 18.4, *N* = 145, Engfors & Churches, 2012). Convergent validity of this task was also established prior to this study by assessing its relationship with the Wechsler Memory III scales of immediate (*r* = 0.67, *p* < .001) and delayed unfamiliar face memory (*r* = 0.69, *p* < .001, *N* = 30). Inter-item reliability of this test for this sample was excellent, *α* = 0.89.

###### 2.1.1.2.2. Social network measures

####### 2.1.1.2.2.1. Sheer size

######## 2.1.1.2.2.1.1. Dunbar’s number (Dunbar, 1993)

Respondents were asked to list the initials of everyone whom they have had social contact with during the last 7 days and the rest of the last month. Contact could consist of any means of interaction, i.e., face-to-face, phone call, email or text message. We used the (free recall) version of this measure from Lewis et al. (2011) administration of the same questionnaire.

######## 2.1.1.2.2.1.2. Social network index (SNI- Number; Cohen, 1997)

The SNI- Number assesses participation in 12 types of social relationships. For example, if the respondent is a parent, they are asked how much contact they have with their children, if a student, how much contact they have with other students. The SNI is a widely used assessment of the size of individuals’ real-life social networks (Liu et al., 2018).

######## 2.1.1.2.2.1.3. Facebook friends

To assess the size of individuals’ online social network participants reported their current number of friends on the social networking site Facebook.

####### 2.1.1.2.2.2. Quality

######## 2.1.1.2.2.2.1. The norbeck social support questionnaire - number (NSSQ-N; Norbeck et al., 1981)

The NSSQ-N is a subscale of the NSSQ that specifically measures the number of people in an individual’s social network who provide personal support. Respondents are asked to list all of the significant people who they can rely on for emotional support, financial assistance, and help with daily tasks etc.

##### 2.1.1.3. Procedure.

This research was approved by the University of Western Australia Human Research Ethics Office, and all participants provided informed consent. Participants were individually tested in a lab setting by a team of experimenters. After obtaining informed consent and basic demographic information, including self-reported sex,^3^ and number of Facebook friends, participants were positioned at approximately 60 cm from a computer screen in a quiet cubicle. The tasks were completed in the following order: CFMT; Dunbar’s number; an Expression task (results reported in the Supplementary Materials); Social Network Index; Famous Faces task and the NSSQ-N. The testing sessions lasted approximately 2 h and ended with a debriefing.

#### 2.1.2. Study 1 results

##### 2.1.2.1. Preliminary analyses.

Five outliers were identified using the inter-quartile outlier-labeling rule with a 3.0 multiplier (Hoaglin & Iglewicz, 1987). Three outliers on the Facebook friends measure (> 2000 friends) and two outliers on Dunbar’s number. Outliers were winsorized by using a value 1% higher or lower than the nearest non-extreme value (Tabachnick & Fidell, 2007). All distributions were suitable for parametric analysis, as skew and kurtosis values met established criteria (skew < |2.0| and kurtosis < |9.0|, Bishara & Hittner, 2012; Edgell & Noon, 1984). Standard deviations indicated a wide range of scores, and mean performance scores showed no floor or ceiling effects. See Table 2 for details. All data in the following analysis are available on the OSF repository ([dataset] Engfors et al., OSF, DATA_Study1_PracticalFR_Social.xlsx, 2023, https://osf.io/4bx7d/).

##### 2.1.2.2. Study 1 main analysis.

Pearson correlations between face recognition ability and SNS measures are presented in Table 3.^4^ To obtain a more precise estimate of the strength of the associations between constructs, we corrected all correlations for the error associated with their measurement (Schmidt & Hunter, 1996, 1999). Throughout the text, we use ‘*r*_*c*_’ to denote these corrected correlations. Tables present the corrected correlations in parentheses and the corresponding uncorrected correlations, along with their associated 95% confidence intervals, which reflect the variability of the estimates. Bayesian Factors for these correlations are available in the Supplementary material and on the OSF repository (Engfors et al., OSF, Bayesian Factors Study 1–4_PracticalFR_Social.docx, 2023, https://osf.io/4bx7d/).

**Fig. 1.**
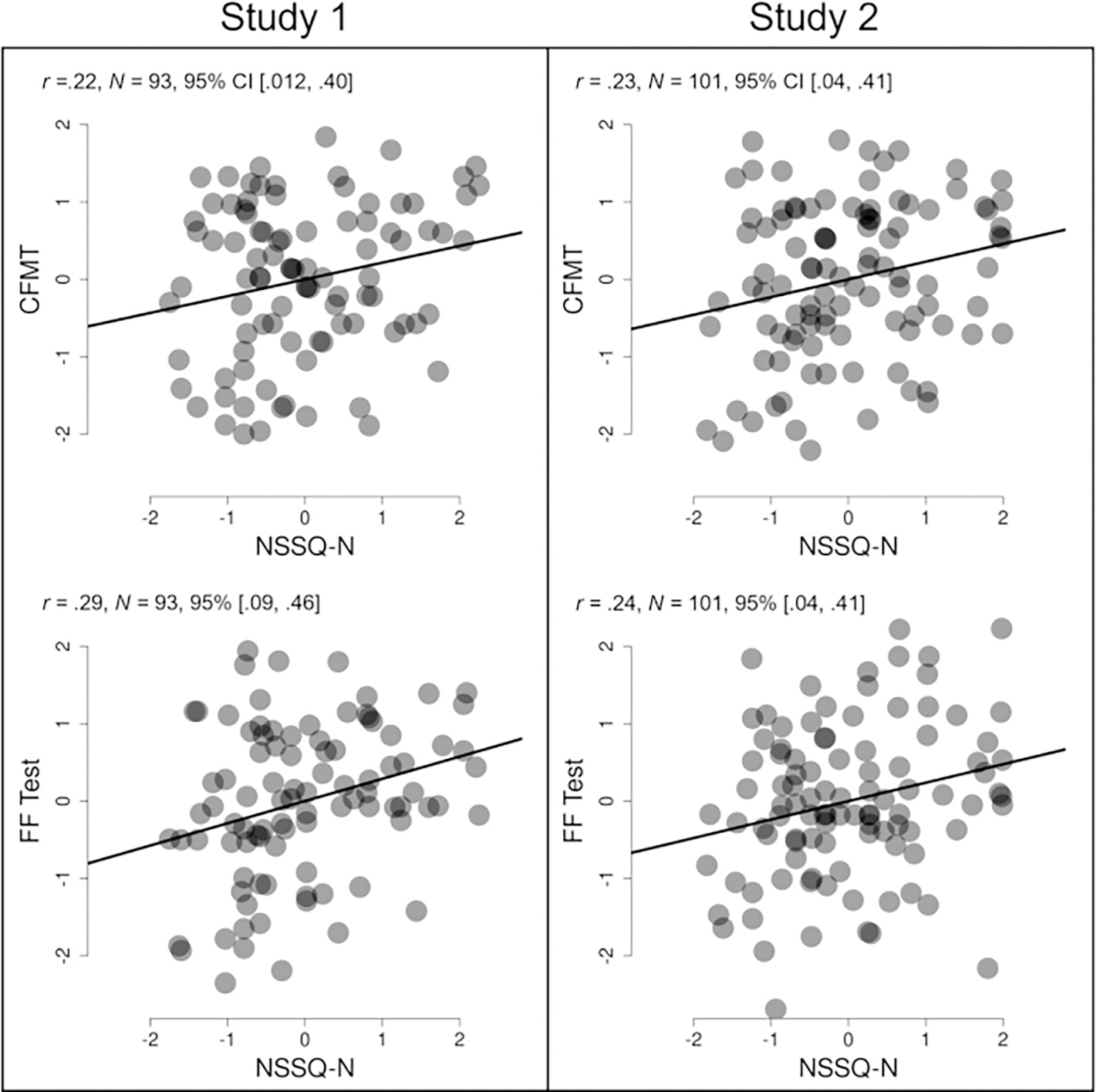
Associations between unfamiliar and familiar face recognition ability and social network quality across Study 1 and 2. *Note.* Graphs were generated using the **Correlation: two measures** app at ShowMyData.org (Wilmer, 2022), which selects axis ranges and aspect ratios such that the physical slope of the regression line exactly equals the correlation coefficient. Scores shown for each test are z-scores after regressing out age and gender. CFMT – Cambridge Face Memory Test; FF Test – Famous Faces Test; NSSQ-N – Norbeck Social Support Questionnaire.

First, we examined whether unfamiliar face recognition ability was correlated with SNS, real-life (SNI and Dunbar’s number) and online (FB friends). They were not significantly associated (SNI; *r*_*c*_ = .03, *p* = .798; Dunbar-N *r*_*c*_ = .01, *p* = .914; FB friends, *r*_*c*_ = −.06, *p* = .579). However, unfamiliar face recognition performance showed a moderate and significant positive correlation with the number of high-quality relationships in one’s network (NSSQ – N) (*r*_*c*_ = .23, *p* = .038), accounting for 5.3% of the reliable variation in the number of high-quality relationships. See Fig. 1 for an illustration of this association.

Next, we estimated the associations between familiar face recognition ability and SNS. Familiar face recognition performance was not significantly correlated with either real-life (SNI: *r*_*c*_ = .07, *p* = .529; Dunbar-N: *r*_*c*_ = −.01, *p* = .931) or online SNS (FB friends, r_c_ = . − .01, *p* = .915). However, as with unfamiliar face recognition, familiar face recognition performance was moderately and significantly associated with possessing a greater number of high-quality relationships (NSSQ – N, *r*_c_ = .31, *p* = .005),^5^ accounting for 9.6% of the reliable variation in the number of high-quality relationships. See Fig. 1 for an illustration of this association.

#### 2.1.3. Study 1 discussion

The results of Study 1 revealed moderate and significant positive correlations between both unfamiliar and familiar face recognition ability and the number of high-quality relationships in one’s social network. Interestingly, we did not observe a significant correlation between either unfamiliar or familiar face recognition ability and the sheer SNS measures. These findings provide the first empirical evidence of a potentially important link between typical face recognition ability and the quality of one’s social network.

### 2.2. Study 2

The aim of Study 2 was to replicate and expand upon the findings from Study 1 using a different sample. We also made two changes: we excluded the Dunbar’s number measure of SNS, due to its unreliability in estimating SNS through free-listing social contacts, and we added the NEO-PI measure of extraversion, which is a commonly used indicator of social experience in previous research on face recognition ability (refer to Table 1). Based on the results of Study 1, our hypothesis for Study 2 posited a significant association between unfamiliar and familiar face recognition ability performance and the number of high-quality social connections in one’s network, However, we did not expect to find a significant association with sheer SNS either in real-life or online. Likewise, we did not expect extraverts to exhibit superior unfamiliar face recognition abilities, consistent with previous research (see Table 1). However, building on the findings of Lander and Poyarekar (2015), See Table 1), we hypothesised that extraverts might demonstrate better memory for familiar (famous) faces. We also expected extraverts to have a larger number of contacts in their social networks, both in real life and online, but not necessarily a greater number of high-quality relationships. This hypothesis aligns with studies by Pollet, Roberts, and Dunbar (2011) and others (Amichai-Hamburger & Vinitzky, 2010; Bolger & Eckenrode, 1991; Kalish & Robins, 2006; Swickert, Rosentreter, Hittner, & Mushrush, 2002).

#### 2.2.1. Study 2 method

##### 2.2.1.1. Participants.

One hundred and one participants between the ages of 18 and 33, were recruited from the University of Western Australia community. All participants reported normal or corrected to normal vision. All data were included in the final analysis, resulting in a final sample of 101 participants (47 males, age range 18–33 years, *M*_age_ = 21.08 yrs., *SD*age = 2.72).

##### 2.2.1.2. Measures

###### 2.2.1.2.1. Face recognition ability measures

####### 2.2.1.2.1.1. Unfamiliar face recognition ability

As in Study 1, we used the CFMT (Duchaine & Nakayama, 2006) to assess unfamiliar face recognition ability, reliability for this sample was excellent: inter-item α = .88.

####### 2.2.1.2.1.2. Familiar (famous) face recognition ability

We used the same famous face recognition test (Engfors & Churches, 2012) used in Study 1. Inter-item reliability for this sample was excellent: α = .90.

###### 2.2.1.2.2. Social network measures

####### 2.2.1.2.2.1. Sheer size

As in Study 1, we used the Social Network Index (SNI-Number; Cohen, 1997) to assess the sheer size of individuals’ real-life social networks and we used Facebook Friends to assess their online social network. To ensure accurate estimates participants were asked to check their Facebook accounts on their phones or a lab computer, at the time of testing.

####### 2.2.1.2.2.2. Quality

To assess the quality of participants’ social networks, we used the Norbeck Social Support Questionnaire-Number (NSSQ-N) as in Study 1 (Norbeck et al., 1981). The NSSQ-N involves the participant listing all individuals who provide them with significant personal support.

####### 2.2.1.2.2.3. Personality trait measure

######## 2.2.1.2.2.3.1. Extraversion

The 47-item Extraversion scale from the NEO-Personality Inventory-Revised (NEO-PIR; Costa & McCrae, 1992, α = .89, *N* = 1539) consists of six lower-order facets, gregariousness, warmth, positive emotions, assertiveness, activity, and excitement seeking. To specifically tap the most social, affiliative aspect of Extraversion we parsed these lower-order facets into two factors: affiliative (represented by gregariousness, warmth and positive emotions, with question such as “I like to have a lot of people around me”; “I really enjoy talking to people) and agentic (represented by assertiveness, activity and excitement seeking, with questions such as, “I have often been a leader of groups I have belonged to”, “When I do things, I do them vigorously”) (Depue, Morrone-Strupinsky, & v., 2005). Inter-item reliability for each of the facets in this sample was very good (affiliative, α =.84, agentic, α =.74; inter-subscale reliability α = .66).

##### 2.2.1.3. Procedure.

This research was approved by the University of Western Australia Human Research Ethics Office, and all participants provided informed consent. All participants were tested individually in the lab by a team of experimenters. After obtaining informed consent and basic demographic information, including self-reported sex, and number of Facebook friends, participants were positioned at approximately 60 cm from a computer screen in a quiet cubicle. The tasks were completed in the following order: CFMT; Social Network Index; Extraversion measure; Famous Faces task; NSSQ-N. The testing sessions lasted approximately 2 h and ended with a debriefing.

#### 2.2.2. Study 2 results

##### 2.2.2.1. Preliminary analyses.

Two outliers were identified using the inter-quartile outlier-labeling rule with a 3.0 multiplier (Hoaglin & Iglewicz, 1987). One outlier on the Facebook friends measure (> 2000 friends) and one outlier for SNI number (54 friends). Outliers were winsorized by replacing them with a value 1% higher or lower than the closest non-extreme value (Tabachnick & Fidell, 2007). As can be seen in Table 4, all distributions were suitable for parametric analysis (skew < | 2.0| and kurtosis < |9.0|, Bishara & Hittner, 2012; Edgell & Noon, 1984). Standard deviations indicated a sufficiently wide range of scores and mean performance scores relative to the maximum and minimum scores also indicated no floor or ceiling effects (see Table 4). All data in the following analysis are available on OSF ([dataset] Engfors et al., OSF, DATA_Study2_PracticalFR_Social.xlsx, 2023, https://osf.io/4bx7d).

##### 2.2.2.2. Study 2 main analysis.

Table 5 presents the Pearson correlations between the measures of face recognition ability, SNS and extraversion in Study 2. Consistent with the findings of Study 1, there were no statistically significant correlations between unfamiliar face recognition performance and sheer SNS in either real-life (SNI; *r*_*c*_ = −0.08, *p* = .422) or online (FB friends, *r*_*c*_ = 0.06, *p* = .525). However, replicating the results of Study 1, unfamiliar face recognition performance was significantly positively correlated with the number of high-quality relationships in one’s network (NSSQ – N) (*r*_*c*_ = 0.26, *p* = .021) accounting for 6.8% of the reliable variation in the number of high-quality relationships. See Fig. 1 for a representation of this correlation.

Next, we examined the associations between familiar face recognition ability and SNS. Consistent with the findings of Study 1, there was a significant positive correlation between familiar face recognition ability and the number of high-quality relationships in one’s network (NSSQ – N, *r*_*c*_ = 0.25, *p* = .017),^6^ accounting for 6.25% of the reliable variation in the number of high-quality relationships. See Fig. 1 for a representation of this correlation. Also, as found in Study 1, familiar face recognition performance was not significantly correlated with one’s real-life SNS (SNI: *r*_*c*_ = 0.20, *p* = .053). However, we did observe a significant positive correlation between familiar face recognition ability and online SNS (FB friends, *r*_*c*_ = 0.32, *p* = .002).^7^ Bayesian Factors for these correlations are available in the Supplementary material and on the OSF repository (Engfors et al., OSF, Bayesian Factors Study 1–4_PracticalFR_Social.docx, 2023, https://osf.io/4bx7d/).

##### 2.2.2.3. Relationships with extraversion.

Table 5 also presents the correlations with extraversion, which align with our expectations. Affiliative extraversion, representing the more sociable aspect of extraversion, was not significantly correlated with unfamiliar face recognition performance (*r*_*c*_ = 0.01, *p* = .920). However, it did show a moderate and significant positive correlation with familiar face recognition performance (*r*_*c*_ = 0.26, *p* = .021). Also, as expected, extraversion was positively and significantly correlated with both the sheer size of one’s social network (SNI, number of people regularly interacted with; *r*_*c*_ = 0.35, *p* = .0005) and the size of one’s online social network (FB friends; *r*_*c*_ = 0.35, *p* = .0005) but not with the number of high-quality relationships (NSSQ – N, *r*_*c*_ = 0.13, *p* = .223).

Based on this pattern of correlations, it was possible that extraversion moderated the correlation between familiar face recognition performance and the sheer SNS measures. To investigate this potential moderation effect, an exploratory moderation analysis was conducted between familiar face recognition performance and SNS (real-life), controlling for affiliative extraversion. There was a significant interaction effect (*β* = −.0.20, *t* = 2.057, *p* = .042), indicating that the correlation between familiar face recognition ability and SNS (real-life) varied depending on levels of affiliative extraversion.

Simple slope analyses were conducted to examine this interaction effect. There was no significant correlation between familiar face recognition performance and SNS for individuals high in affiliative extraversion (*β* = −0.087, *t* = −0.297, *p* = .771), however, there was a significant positive correlation for individuals low in affiliative extraversion (*β* = 0.610, *t* = 2.865, *p* = .013). This pattern of results suggests that the correlation between familiar face recognition ability and social network size may only be apparent for introverts (that is introverts with poor familiar face recognition abilities tend to have smaller social networks). In contrast, the relationship was weaker or non-existent for individuals high in affiliative extraversion. We also conducted a moderation analysis between familiar face recognition performance and online (FB friends), controlling for affiliative extraversion; however the results revealed no significant interaction effect (*β* = 0.04, *t* = 0.372, *p* = .710) so we did not examine this further.

### 2.3. Studies 1 and 2 discussion

Consistently across two independent studies, we observed significant and moderate correlations (in line with the guidelines for interpreting effect sizes in individual differences research proposed by Gignac & Szodorai, 2016) between both unfamiliar and familiar face recognition abilities and a greater number of high-quality relationships in one’s network. These findings provide the first evidence of an association between face recognition abilities and the presence of meaningful social connections in the general population. However, we did not find convincing evidence for a consistent relationship between face recognition abilities and the quantity of social connections. Future research with larger samples will be needed to determine if there may exist a small relationship with social network size alone that the present series of studies was unable to reliably detect. Additionally, preliminary evidence in Study 2 suggests a potential link between familiar face recognition ability, SNS, and levels of affiliative extraversion with introverts with poor familiar face recognition abilities tending to have smaller social networks. These findings provide an initial indication that extraverts may rely on other social skills beyond face recognition ability to expand their social networks. However, further research is needed to replicate and validate these results.

Overall, these findings suggest that face recognition ability may play a key role in the development and maintenance of meaningful social connections. Individuals with better face recognition abilities may be better equipped to navigate social interactions, recognise familiar individuals, and form strong bonds with others. However, the relationship between face recognition abilities and social networks may be complex and modulated by other individual differences, such as extraversion. Further research is needed to explore these relationships and their implications for social functioning and well-being.

## 3. Studies 3 and 4

The aim of the next two studies was to provide further insight into the connection between face recognition ability and social experience, with a specific focus on various personality traits related to social interaction. Previous investigations have primarily relied on extraversion as a proxy for social experience and have yielded limited evidence of its involvement (refer to Table 1). However, the previous studies may have been constrained by methodological limitations, such as exclusive reliance on internet-based data collection, absence of controlled laboratory settings, or inadequate sample sizes to detect subtle yet meaningful effects. Another possibility is that the association between extraversion, akin to sheer social network size, and unfamiliar face recognition abilities may be minimal. With these two possibilities in mind, we designed the following two studies with a specific emphasis on addressing the limitations of prior research.

To achieve this, we employed rigorous methodology in a controlled laboratory environment, comprehensive measures, and a large sample size, enabling a comprehensive exploration of this relationship. In Study 3, we employed a stringent research design in a controlled laboratory setting with each participant tested individually by the experimenter. We isolated unfamiliar face recognition ability from more general visual recognition ability by including an object recognition test, and the face-specific component of face recognition performance was isolated using residuals (DeGutis, Wilmer, Mercado, & Cohan, 2013). We examined their correlation with a comprehensive assessment of extraversion, along with a measure of sociability, the need to belong, and measures assessing social difficulty. Then, in Study 4, we recruited the largest sample to date (*N* = 2000+) examining the correlation between face recognition ability and extraversion. We recruited participants via the web and focused specifically on extraversion and its association with unfamiliar face recognition ability.

While we did not anticipate a substantial correlation between personality traits related to social interaction, such as extraversion, and unfamiliar face recognition ability, Study 3 and 4 were designed to offer valuable insights into the possible interplay between personality traits related to social interaction and face recognition ability. By doing so, our goal was to advance our understanding of the connection between face recognition and social experience.

### 3.1. Study 3

#### 3.1.1. Study method

##### 3.1.1.1. Participants.

Two hundred and four Caucasian undergraduate students from the University of Western Australia participated for course credit. All participants underwent vision testing, to ensure normal or corrected to normal vision. Due to a computer malfunction, data for one participant was lost, hence we had a dataset of 203 for analysis (81 males, age range 17–32 yrs., *M*_age_ = 19.63 yrs., *SD*_age_ = 2.79).

##### 3.1.1.2. Measures

###### 3.1.1.2.1. Recognition ability measures

####### 3.1.1.2.1.1. Unfamiliar face recognition ability

Consistent with all the studies presented in this paper, we used the CFMT (Duchaine & Nakayama, 2006) to measure unfamiliar face recognition ability. Inter-item reliability for this sample, *α* = 0.88.

####### 3.1.1.2.1.2. Object recognition ability

To isolate face recognition ability from general visual memory, we included the Cambridge Car Memory Test (CCMT; Dennett et al., 2012). The CCMT test is identical in format to the CFMT but uses car images instead of faces. Reliability of the CCMT test is also excellent, inter-item reliability for this sample, *α* = 0.86.

####### 3.1.1.2.1.3. Face-selective ability

To control for any variance associated with general visual memory, we created a “face-selective ability” variable using the residuals from a first-order regression, predicting CFMT scores from CCMT scores. Face-selective reliability (based on Malgady & Colon-Malgady’s, 1991 formula) for this sample was good *α* = 0.75.

###### 3.1.1.2.2. Personality trait measures

####### 3.1.1.2.2.1. Extraversion

We again used the NEO-PIR (Costa & McCrae, 1992), and as in Study 2, we focused on the two lower order affiliative and agentic facets to isolate the most social aspect of extraversion (Depue et al., 2005). Reliability for this sample was excellent, *α* = 0.91 and *α* = 0.81, respectively.

####### 3.1.1.2.2.2. Sociability

A 5-item measure of Sociability (Cheek & Buss, 1981) was used to assess enjoyment in social interaction. Sample items include, “I welcome the opportunity to mix socially with people,” and “I’d be unhappy if I were prevented from making many social contacts.” Reliability for the current sample was excellent (*α* = 0.86).

####### 3.1.1.2.2.3. The need to belong

A 10-item scale (Leary, Kelly, Cottrell, & Schreindorfer, 2013) was used to assess the extent to which people need to feel socially connected. Sample items from this scale are, “I do not like being alone” and “It bothers me a great deal when I am not included in other people’s plans.” The measure had excellent reliability (*α* = 0.86).

####### 3.1.1.2.2.4. Social difficulty

######## 3.1.1.2.2.4.1. Autistic traits

The widely used Autism-Spectrum Quotient (AQ; Baron-Cohen, Wheelwright, Skinner, Martin, & Clubley, 2001) was used to assess autistic traits in the general population. To specifically tap social difficulties, we focused on the “Social Skills” factor based on a four-factor solution (Russell-Smith, Maybery, & Bayliss, 2011), which includes 12-items such as “I enjoy social occasions” (negatively scored). Higher scores on this scale indicate greater social difficulties; hence, we refer to the social skills factor as autistic trait linked “social difficulties” to prevent confusion. Inter-item reliability for the social skills factor was very good in this sample (*α* = 0.79), with reliability for the other 3 factors poorer (“Details”, 9 items, *α* = 0.45, “Imagination”, 6 items, *α* = 0.33 and “Understanding”, 10 items, *α* = 0.47).

######## 3.1.1.2.2.4.2. Social anxiety

The 20-item Social Interaction Anxiety Scale (Mattick & Clarke, 1998, *α* = 0.94, *N* = 1069) measures anxiety regarding social interactions. Sample items from this measure are, “I have difficulty making eye contact with others” and “I have difficulty talking with other people.” The measure displayed excellent reliability in this sample (α = 0.93).

##### 3.1.1.3. Procedure.

All participants completed the study individually with the same experimenter. After providing informed consent and demographic information, participants were seated in a quiet cubicle approximately 60 cm from a computer screen. Tasks were administered in the following sequence: composite effect task, CFMT, Extraversion and Need to Belong questionnaires, face identity aftereffect task, expression labeling task, Social Anxiety, Sociability and AQ questionnaires. The CCMT was administered last, followed by a debriefing. The entire session lasted approximately 2 h. This study was approved by The University of Western Australia’s Human Research Ethics Office and results of the composite and identity aftereffect and their correlation with face recognition ability have been reported previously in Engfors et al. (2017). The expression labeling task was peripheral, and the correlations with the self-report questionnaires were not considered noteworthy, but for completeness, they are provided in the Supplementary Materials.

#### 3.1.2. Study 3 results

##### 3.1.2.1. Preliminary analyses.

No univariate outliers were identified and as can be seen in Table 6, all distributions were suitable for parametric analysis (skew < |2.0| and kurtosis < |9.0|, Bishara & Hittner, 2012; Edgell & Noon, 1984). Standard deviations indicated a sufficiently wide range of scores and mean performance scores relative to the maximum and minimum scores also indicated no floor or ceiling effects (see Table 6). All data in the following analysis are available on OSF ([dataset] Engfors et al., OSF, DATA_Study3_PracticalFR_Social.xlsx, 2023, https://osf.io/4bx7d/).

##### 3.1.2.2. Study 3 main analysis.

Table 7 presents the Pearson correlations among sociable personality traits, unfamiliar face recognition performance, and face-selective performance. We did not observe any significant positive correlations between sociable personality traits and face recognition ability. For example, the correlation between affiliative extraversion and unfamiliar face recognition performance (i.e. CFMT) (*r*_*c*_ = 0.04) and face recognition ability controlling for non-face recognition ability (i.e. face-selective performance) (*r*_*c*_ = 0.07) were not statistically significant (*p* = .645 and, *p* = .395, respectively). The corrected correlations for our other personality measures of sociability and unfamiliar face recognition performance ranged from *r*_*c*_ ≈ 0.00 for the Need to Belong to *r*_*c*_ ≈ 0.08 for Sociability. As expected, all sociable personality trait measures demonstrated high convergence, indicating they measured a similar construct. Additionally, sociability and affiliative extraversion were strongly and negatively correlated with autism-like social difficulties (*r*_*c*_ = −0.66, *r*_*c*_ = −0.66, respectively) and social anxiety (*r*_*c*_ = −0.58, *r*_*c*_ = −0.70, respectively), also as expected (Rodebaugh, Woods, & Heimberg, 2007; Wakabayashi, Baron-Cohen, & Wheelwright, 2006).

We also examined the possibility of a negative correlation with face recognition ability and social difficulties (as assessed by social anxiety and autism-like social difficulties). Despite not achieving statistical significance, the effects observed were comparable in magnitude to previous studies. For example, the effect size observed in our study (*r*_*c*_ = −0.16) for the association between face-selective recognition and social anxiety was similar to the effect sizes reported by Davis et al. (2011; *r* (137) = −0.18) and Palermo et al. (2017; *r*(136) = −0.22). Likewise, the effect size (*r*_*c*_ = −0.13) found between face-selective recognition and autism-like social difficulties aligned with prior research that used a slightly different social difficulties AQ factor structure (Davis et al., 2017; *r*(90) = −0.14). Bayesian Factors for these correlations are available in the Supplementary material and on the OSF repository (Engfors et al., OSF, Bayesian Factors Study 1–4_PracticalFR_Social.docx, 2023, https://osf.io/4bx7d/).

#### 3.1.3. Study 3 discussion

In Study 3, conducted in a highly-controlled lab environment, we observed small, non-significant correlations between unfamiliar face recognition ability and sociable personality traits, including extraversion and sociability. Given the potential for subtle effects that may not have been detected in our initial sample of 200 participants, we extended our investigation in Study 4. Using a larger data set of over 2000 participants, recruited online, we specifically concentrated our analysis on the association between extraversion and unfamiliar face recognition performance. This larger sample size offered an additional, robust evaluation of any potentially significant association.

### 3.2. Study 4

#### 3.2.1. Method

##### 3.2.1.1. Participants.

A dataset containing the data of 2696 participants was sourced via TestMyBrain.org, a digital research platform where participants complete online tests in exchange for their personalised results (Germine et al., 2012). We excluded participants who neglected to report either age or sex (*n* = 71), who reported an age outside of the range 10–70 (*n* = 22), or who reported being raised in a country that was not predominantly Caucasian (*n* = 569). This resulted in a dataset of *N* = 2028 for the analysis (585 males, age range 10–69 years, *M*_*age*_ = 24.80 yrs., *SD*_*age*_ = 11.92).

##### 3.2.1.2. Measures

###### 3.2.1.2.1. Personality trait measure

####### 3.2.1.2.1.1. Extraversion

The self-report Big Five Inventory (BFI; John, Donahue, & Kentle, 1991) was used to assess Extraversion, as well as four other personality dimensions: Openness, Conscientiousness, Agreeableness, and Neuroticism. This 44-item measure uses a 5-point Likert scale and is a widely used, well-validated and reliable measure (inter-item reliability coefficient for Extraversion is 0.88; John & Srivastava, 1999; Rammstedt & John, 2007). Reliability estimates for the current sample: Extraversion *α* = 0.85, Openness *α* = 0.69, Conscientiousness *α* = 0.83, Agreeableness *α* = 0.74 and Neuroticism *α* = 0.83.

####### 3.2.1.2.1.2. Face recognition measure

######## 3.2.1.2.1.2.1. Unfamiliar face recognition ability

The CFMT (Duchaine & Nakayama, 2006) was used. Reliability was excellent (for this sample: inter-item, *α* = 0.90). Prior studies have shown comparable data quality between lab and online versions of CFMT completed on TestMyBrain.org (Germine et al., 2012).

##### 3.2.1.3. Procedure.

Participants were voluntary visitors to the TestMyBrain.org website that was openly available for anyone, during 2014–2015. As participation is completely voluntary, no compensation was provided, and participants were free to terminate their involvement at any point. A test battery comprising the extraversion measure and face recognition ability test was featured on the site under the title ‘Personality, Faces, and Words’. The experiment commenced with an explanation of the study, followed by participants providing consent to proceed. Participants then responded to demographic questions including age, sex, and whether English was their first language. Subsequently, participants completed the five BFI subtests, in the following order: openness, conscientiousness, neuroticism, agreeableness, and extraversion. This was followed by a brief questionnaire regarding their current life experiences before taking the Cambridge Face Memory Test (CFMT) and a Vocabulary test. The session concluded with a final set of demographic questions. All study and informed consent procedures were reviewed by the Committee on the Use of Human Subjects at Harvard.

#### 3.2.2. Study 4 results

##### 3.2.2.1.1. Preliminary analyses.

No influential univariate outliers were identified and as shown in Table 8 all distributions were suitable for parametric analysis (skew < |2.0| and kurtosis < |9.0|, Bishara & Hittner, 2012; Edgell & Noon, 1984). Standard deviations indicate a sufficiently wide range of scores for individual differences analyses and mean performance scores relative to the maximum and minimum scores also indicated no floor or ceiling effects (also see Table 8). All data in the following analysis are available on OSF ([dataset] Engfors et al., OSF, DATA_Study4_PracticalFR_Social.xlsx, 2023, https://osf.io/4bx7d/).

##### 3.2.2.2. Main analysis.

Table 9 presents the Pearson correlations between unfamiliar face recognition ability and the five personality traits of the BFI. As in Study 3, we examined the potential positive correlation between social experience, as measured by the Extraversion subscale of the BFI, and unfamiliar face recognition performance, as assessed by the CFMT. The correlation was small, though, given the large sample size, it was close to statistically significant (*r*_*c*_ = 0.05, *p* = .074). The magnitude of this effect was comparable to that observed in Study 3 between unfamiliar face recognition ability and affiliative extraversion (*r*_*c*_ = 0.04). There were no other noteworthy correlations; however, we did observe a significant positive correlation between unfamiliar face recognition ability and Openness (*r*_*c*_ = 0.09, *p* = .002). Notably, the BFI-Openness questionnaire includes items related to artistic interests, which previous research suggests may be associated with face perception skills (e.g., Devue & Barsics, 2016). In addition, Openness has been linked to general cognitive ability (Gignac, Stough, & Loukomitis, 2004), which, although to a small extent, is also correlated with face recognition (Gignac, Shankaralingam, Walker, & Kilpatrick, 2016; Wilmer, 2017). These factors may contribute to the observed small correlation. Bayesian Factors for these correlations are available in the Supplementary material and on the OSF repository (Engfors et al., OSF, Bayesian Factors Study 1–4_PracticalFR_Social.docx, 2023, https://osf.io/4bx7d/).

### 3.3. Study 3 and 4 discussion

We conducted two comprehensive studies using reliable, validated measures to investigate the potential link between unfamiliar face recognition ability and sociable personality traits, including the widely recognised trait of extraversion. In our initial meta-analysis of five studies (see Table 1) we observed a small meta-analytic effect size of *r* = 0.11 (95% CI [0.06, 0.16]) between unfamiliar face recognition ability and extraversion. Our subsequent findings consistently demonstrated an even smaller association between these variables, with a mean correlation of *r* = 0.04 (95% CI [0.00, 0.08]). In light of Kvarven et al. (2019) findings on the potential for nearly threefold inflation of mean-analytic effect sizes in studies of human behaviour, our replicated correlation of *r* = 0.04 likely provides a more accurate estimate of the association between face recognition ability and extraversion. While correlation does not equal causation, the lack of correlation does imply a lack of causation. As such, the very small associations we found would suggest that any causal link between face recognition ability and extraversion would be very modest. Similarly, we find little to no correlation of unfamiliar face recognition with an array of other sociability-related trait measures in Study 3.

## 4. General discussion

Recent years have seen a surge in research focused on individual differences in face recognition ability, largely driven by the potential connection between variation in this ability and real-world social experiences. However, despite this interest, there is limited evidence supporting this association. To bridge this gap, we conducted four studies that examined the relationship between face recognition and various measures of social experience, within the typical population. In Studies 1 and 2, we found, and then replicated, an association between face recognition abilities (both unfamiliar and familiar) and the number of high-quality relationships reported. Interestingly, this association did not extend to sheer quantity of one’s social network, that is, estimates of overall social network size which gauge interaction with acquaintances and friends. Nor did it extend to extraversion, a personality trait closely linked with having greater numbers of social connections (Studies 3 and 4).

These findings are important because they provide evidence to support a longstanding, under-examined assumption that face recognition abilities are linked with social experience in the general population. However, our results also indicate the link is more nuanced than broad assumptions may suggest. Our findings showed a specific link between face recognition and the quality, not sheer quantity, of one’s social connections. The relative absence of a link to the quantity of social connections challenges both the assumption that a benefit of good face recognition ability is that it facilitates large social networks and the assumption that interaction with many people necessarily enhances face recognition skills. Rather our findings suggest a more nuanced understanding of the practical relevance of face recognition abilities in everyday social experiences within the non-clinical population. They further highlight the need for more varied empirical investigation to better understand the practical correlates of face recognition ability and the causal mechanisms involved. An important next step will be for researchers to establish the generalisability of our findings by determining the degree to which the relationship we discovered between face recognition ability and social relationship quality extends to different measures of quality, face recognition ability, and to different populations. Given the modest size of the association it will be important to ensure such investigations are well-powered and the measures are psychometrically robust.

Although our research does not claim to establish a causal link, it does raise questions about the possible causal relationships between face recognition ability and the number of high-quality social connections. For example, though our results suggest that mere exposure to many more superficial social contacts may have minimal effects, it remains plausible that accumulating experiences from many close, supportive ties over time might refine and enhance initial face recognition skills. In particular, extensive experience with the same individuals across changing contexts and over many encounters could aid in building robust representations resilient to intra-person variability (Ritchie & Burton, 2017)—a skill that could transfer more broadly (Abudarham, Shkiller, & Yovel, 2019). This possibility also aligns with the Social Motivation Theory of Autism (Chevallier, Kohls, Troiani, Brodkin, & Schultz, 2012), which proposes reduced social reward motivation cascades into the selective impairments in face recognition seen in this population. Alternatively, strong face recognition abilities could facilitate the formation and maintenance of meaningful relationships from the outset, whereas poorer abilities could conversely limit or constrain relationship-building opportunities and attainment of intimate social connections.

To the extent that a solid capacity to recognise faces does have practical value, for the development of high-quality social connections, or for other social or non-social reasons that have yet to be explored, the question arises: are there steps that can or should be taken to aid or support people in their recognition of others? In theory, one or both of two broad approaches might be taken to provide such support.

On the one hand, one might seek to intervene in the individual themselves to improve their face recognition. In the training studies undertaken to date, however, while focused skills improved, they tended not generalise beyond the laboratory to everyday life, at least in individuals with clinically poor face recognition abilities (see Bate & Bennetts, 2014 for a review). These findings complement evidence from twin studies that environmental factors contribute relatively little to the capacity to recognise commonly experienced face types (Wilmer, 2017; Wilmer et al., 2010). It nevertheless remains possible that a yet-to-be-discovered intervention—sufficiently strong, targeted, early, or sustained—could have a positive impact. For example, conceivably, given our results, an intervention that targets, or mimics, the initiation and maintenance of early, high-quality social relationships could have a positive impact on face recognition ability.

Another broad approach to aiding recognition is to intervene in the environment to make it more supportive of a diverse range of face recognition capacities. Some such interventions are simple, cheap, and focused, such as the provision of name tags/tents at group gatherings: classes, parties, conferences, or business meetings, for example. Such interventions have been shown to aid those with poor face recognition (Adams, Hills, Bennetts, & Bate, 2020), and they additionally support those who have had less prior opportunity to get to know the people at a gathering. Other effective interventions include assistive technology, such as facial recognition software on smartphones or smartwatches (Adams et al., 2020). Over time, broader efforts to educate the public about the existence of variable face recognition capacities might have additional benefits. On the individual level, they might facilitate self-awareness and self-advocacy. On the public level, they might enhance patience with, and tolerance of, recognition failures.

Our research also provides preliminary evidence that social skills could act as a buffer against the impacts of poor face recognition abilities. Specifically, our exploratory moderation analysis in Study 2 revealed that poor face recognition abilities were associated with smaller social networks, but only among introverted individuals and not among extraverts. This early evidence suggests that strengthening social skills could assist individuals in overcoming their face recognition challenges, leading to the establishment of more social connections. However, it is important to note that the size of an individual’s social network does not guarantee the presence of more meaningful relationships. While social skill training may contribute to expanding one’s social network, the quality and depth of the relationships formed within that network are not solely dependent on social skills. This finding aligns with previous research (Molho et al., 2016; Pollet et al., 2011) and is further supported by the experiences of extraverts in our study. Nonetheless, social skills training remains a promising avenue for enhancing the lives of individuals with poor face recognition abilities. These findings emphasise the necessity for further investigation into the factors that contribute to the development of meaningful relationships in individuals with poor face recognition abilities.

Ultimately, further research is needed to determine the causal relationship between face recognition skills and meaningful social connections. Longitudinal studies that monitor individuals’ face recognition skills and social connections over time could shed light on whether face recognition skills have a causal effect on high-quality social connections or whether social connections drive the development of face recognition ability. Additionally, investigating the potential benefits of compensatory strategies that aim to improve identity recognition across the entire face recognition continuum, rather than just for those with very poor face recognition skills, could enhance the social experience and number of quality relationships for a broader range of individuals. Randomised controlled trials comparing the social experiences and connections of individuals in compensatory training programs to those in control groups could determine the effectiveness of such strategies. Understanding this relationship could have significant practical implications for individuals with poor face recognition skills and could inform the development of targeted interventions to improve social connections in this population.

In conclusion, our series of studies provide the most comprehensive investigation to date into the everyday practical relevance of individual differences in face identity recognition abilities. The results establish a link between face identity recognition ability and social experience. At the same time, they suggest that it is not driven by the extent to which someone enjoys socialising, nor merely by the sheer number of social connections they maintain. Instead, we find that face identity recognition ability is consistently linked to the number of *high-quality* social connections. These results provide important insights into the complex interplay between cognitive and social processes and highlight the need for continued efforts to understand individual differences in face identity recognition ability. Ultimately, our findings have practical implications for enhancing social support and the development of targeted interventions aimed at supporting individuals with face recognition difficulties.

## Supplementary Material

Supplement - Appendix A

## Figures and Tables

**Table 1:** Summary of published studies on the association between face recognition memory ability and extraversion.

Reference	N	Measure	Index	Face Recognition Ability (FRA) test

(Kramer, Hardy, & Ritchie, 2020)	97 (85 f, *M*_age_ = 20.25, *SD*_*a*ge_ = 4.11)	Extraversion from the Mini-IPIP	CFMT	*Extraversion and CFMT: r = .13 [–−.12, .37]*
(Lander & Poyarekar, 2015)	100 (52 f, 18–32 *yrs*; *Med*_age_ = 23)	TIPI 2 Q = Extraversion, plus A; C; ES; O Extra Q’nnaire (EPQ) adapted from personalitytest.net 20 items.	Famous Faces test, faces degraded, and some inverted (M = 50.3, SD = 18.29 upright trials).	*Extraversion (TIPI) & famous FR: r = .23, p = .02* *Extraversion (EPQ) & famous FR: r = .29, p = .003*
(Li et al., 2010)	399 (200 f, M_age_ = 20.2, SD_age_ = 0.9)	Extraversion from the NEO-PI-R	Face recognition ability (FRA): Difference between an unpublished old/new face recognition test and a flower recognition test.	*Extraversion with FRA: r = .09 [−.01–.19]* *Gregariousness with FRA: r = .10 [.00–.20]*
(McCaffery, Robertson, Young, & Burton, 2018)	103 (52 f, *M*_age_ = 53, *SD*_Age_ = 15)	Extraversion from the Big Five	CFMT	*Extraversion and CFMT: r* = *.00 [−.19, .19]*
(Satchell, Davis, Julle-Daniere, Tupper, & Marshman, 2019)	792 (476 f, *M*_age_ = 33.55, *SD*_Age_ = 10.15)	Extraversion from the Big Five	CFMT+	*Extraversion and CFMT+: r = .13 [.06, .20]*
(Sunday, Patel, Dodd, & Gauthier, 2019)	90 (61 f, *M*_age_ = 20.84, *SD*_Age_ = 2.82)	Extraversion from the IPIP	CFMT+	*Extraversion and CFMT+: r = .11 [−.10, .31]*

**Table 2:** Study 1: Descriptive statistics of measures for face recognition ability and social network size (N = 93).

	Min	Max	Mean	*SD*	Skew	Kurtosis

CFMT%	55.56	100.00	78.87	11.64	–−0.33	−0.86
FF%	5.56	89.66	51.42	19.58	−0.31	−0.48
SNI	7	45	24.90	7.96	0.29	−0.01
Dunbar - N	3	142	57.46	30.14	0.75	0.38
FB	80	1450	634.53	328.58	0.77	0.25
NSSQ - N	5	24	12.96	4.95	0.52	−0.48

Note. CFMT% = Cambridge Face Memory Test % correct; FF% = Famous Faces Test % correct of those known; SNI = Social Network Index; FB = Facebook friends; NSSQ-N = Norbeck Social Support Questionnaire.

**Table 3:** Study 1: Intercorrelations (corrected correlations in parentheses) among measures of face recognition ability and social network size, age controlled for, with 95% confidence intervals above the diagonal (N = 93).

	CFMT%	FF%	SNI	Dunbar-N	FB	NSSQ-N	Sex

1. CFMT%	–	[.44–.73]	[−.18–.23]	[−.20–.21]	[−.26–.15]	[.02–.40]	[−.13–.28]
2. FF%	**.62 (.69)**	–	[−.14–.27]	[−.21–.20]	[−.21–.19]	[.09–.46]	[.08–.46]
3. SNI	.03 (.03)	.07 (.07)	–	[−.25–.16]	[−.04–.36]	[.16–.52]	[−.20–.21]
4. Dunbar-N	.009 (.01)	−.01 (−.01)	−.05	–	[−.08–.32]	[−.15–.25]	[−.22–.19]
5. FB	−.06 (−.06)	−.01 (−.01)	.17	.13	–	[−.08–.32]	[−.22–.19]
6. NSSQ-N	**.22 (.23)**	**.29 (.31)**	**.36**	.05	.10	–	[.21–.55]
7. Sex	.08 (.08)	**.28 (.30)**	.00^a^	−.01	.01^a^	**.39**	–

Note. CFMT% = Cambridge Face Memory Test % correct; FF% = Famous Faces Test % correct of those known; SNI = Social Network Index; FB = Facebook friends; NSSQ-N = Norbeck Social Support Questionnaire. Correlations in bold were statistically significant (*p* > .05). All variables are residuals, after controlling for age. Reliability for residuals: CFMT% α = 0.89; FF% α = 0.90; Extraversion α =0.84; Affiliative Extraversion, α = 0.85, Agentic Extraversion, α =0.69.

a Internal consistency reliability could not be calculated for other variables so was set to 1 (perfect reliability). We had no a priori prediction regarding sex, but report here for completeness.

**Table 4:** Study 2: Descriptive statistics of measures for face recognition ability, social network size and extraversion (N = 101).

	Min	Max	Mean	*SD*	Skew	Kurtosis	α

CFMT%	55.56	100.00	80.06	11.15	−.29	−.73	.89
FF%	5.56	93.10	53.26	17.91	−.06	−.13	.89
SNI	4	41	22.58	8.12	.31	−.40	–
FB	50	1112	486.75	233.35	.60	.06	–
NSSQ - N	4	24	13.61	5.28	.37	−.67	–
Total Extraversion	65	148	110.15	16.50	−.27	.17	.85
Affiliative Extraversion	34	79	59.14	9.91	−.11	−.52	.84
Agentic Extraversion	23	73	51.01	9.37	−.36	.68	.74

Note. CFMT% = Cambridge Face Memory Test percentage correct; FF% = Famous Faces Test percentage correct of those known; SNI = Social Network Index; FB = Facebook friends; NSSQ-N = Norbeck Social Support Questionnaire.

**Table 5:** Study 2: Intercorrelations (corrected correlations in parentheses) among measures of face recognition ability, social network size and extraversion, age controlled for, with 95% confidence intervals above the diagonal (N = 101)

Measures	CFMT	FF	SNI	FB	NSSQ	Extra	Affiliative	Agentic	Sex

1. CFMT%	–	[.26–.58]	[−.27–.12]	[−.13–.26]	[.04–.41]	[−.20–.19]	[−.17–.22]	[−.24–.15]	[−.17–.22]
2. FF%	**.43 (.48)**	–	[−.003–.37]	[.12–.47]	[.04–.41]	[.04–.41]	[.04–.41]	[−.03–.36]	[.03–.41]
3. SNI	−.08 (−.08)	.19 (.20)	–	[.13–−0.48]	[.07–.43]	[.14–.49]	[.12–.48]	[.05–.42]	[−.17–.22]
4. FB	.06 (.06)	**.30 (.32)**	**.32**	–	[−.08–.31]	[.12–.48]	[.06–.43]	[.08–.45]	[−.22–.17]
5. NSSQ-N	**.23 (.26)**	**.24 (.25)**	**.26**	.12	–	[−.06–.32]	[−.07–.31]	[−.09–.29]	[−.02–.36]
6. Extraversion	−.01 (−.01)	**.23 (.27)**	**.32 (.35)**	**.31 (.34)**	.13 (.14)	–	[.81–.91]	[.78–.90]	[−.11–.28]
7. Affiliative	.03 (.03)	**.23 (.26)**	**.31 (.34)**	**.26 (.28)**	.12 (.13)	**.87 (1.00)**	–	[.31–.61]	[−.04–.35]
8. Agentic	−.05 (−.06)	.17 (.22)	**.24 (.29)**	**.28 (.34)**	.10 (.12)	**.85 (1.00)**	**.48 (.63)**	–	[−.21–.18]
9. Sex	.02 (.02)	**.23 (.24)**	.02^a^	−.02^a^	.18^a^	.09 (.10)	.16 (.17)	−.02 (−.02)	–

Note. CFMT% = Cambridge Face Memory Test % correct; FF% = Famous Faces Test % correct of those known; SNI = Social Network Index; FB = Facebook friends; NSSQ-N = Norbeck Social Support Questionnaire. Correlations in bold were statistically significant (*p* > .05). All variables are residuals, after controlling for age. Reliability for residuals: CFMT% α = 0.89; FF% α = 0.90; Extraversion α =0.84; Affiliative Extraversion, α = 0.85, Agentic Extraversion, α =0.69.

a Internal consistency reliability could not be calculated for other variables so was set to 1 (perfect reliability). We had no a priori prediction regarding sex, but report here for completeness.

**Table 6:** Study 3: Descriptive statistics of measures for face recognition ability and personality traits (N = 203).

Measures	Min	Max	Mean	*SD*	Skew	Kurtosis	α

CFMT%	52.78	100.00	80.36	11.27	−0.45	−0.54	0.87
CCMT%	37.50	100.00	71.91	12.91	0.08	−0.42	0.86
Total Extraversion	47.00	164.00	117.07	21.44	−0.49	0.06	0.92
Affiliative Extraversion	19.00	89.00	63.21	13.21	−0.67	0.44	0.91
Agentic Extraversion	25.00	79.00	53.91	10.68	−0.34	−0.09	0.81
Sociability	1.00	20.00	12.19	4.08	−0.37	−0.24	0.86
Need to Belong	12.00	48.00	33.66	6.80	−0.48	0.25	0.86
Social Anxiety	1.00	59.00	23.21	12.68	0.68	0.07	0.93
AQ – Social Difficulties	12.00	42.00	23.89	5.66	0.37	−0.06	0.79

Note. AQ = Autism Quotient, *n* = 181 (AQ data was collected separately as part of a broader screening session and was not available for all participants); All other tests *n* = 203.

**Table 7:** Study 3: Intercorrelations (corrected correlations in parentheses) among measures of face recognition ability and personality traits, age controlled for, with 95% confidence intervals above the diagonal (N = 203).

Measures	1	2	3	4	5	6	7	8	9	10	11

1. CFMT%	–	[.93, .96]	[.18, .43]	[−.11, .17]	[−.10, .18]	[−.12, .16]	[−.06, .22]	[−.15, .12]	[−.26, .01]	[−.24 .05]	[−.15, .12]
2. Face Sel.	**.95 (1.00)**	–	[−.14, .14]	[−.10, .18]	[−.08, .20]	[−.14, .14]	[−.05, .22]	[−.14, .14]	[−26, .01]	[−.23 .04]	[−.10, .18]
3. CCMT%	**.31 (.36)**	.00 (.05)	–	[−.14, .14]	[−.19, .09]	[−.08, .20]	[−.15, .13]	[−.20, .08]	[−.15, .13]	[−.16, .12]	[−.30, −.03]
4. Tot. Ext.	.03 (.03)	.04 (.00)	−.00 (−.00)	–	[.90, .94]	[.83, .90]	[.68, .80]	[.09, .34]	[−.72, −.56]	[−.58, −.35]	[−.02, .25]
5. Affil. Ext.	.04 (.04)	.06 (.07)	−.05 (−.06)	**.92 (1.00)**	–	[.52, .69]	[.76, .85]	[.20, .45]	[−.71, −.55]	[−.65, −.46]	[.07, .34]
6. Agent. Ext.	.02 (.02)	.00 (.00)	.06 (.07)	**.87 (1.00)**	**.61 (.71)**	–	[.39, .60]	[−.11, .17]	[−.61, −.41]	[−.39, −.14]	[−.18, .10]
7. Sociability	.08 (.08)	.09 (.11)	−.01 (−.01)	**.75 (.84)**	**.81 (.92)**	**.50 (.60)**	–	[.25, .48]	[−.61, −.41]	[−.63, −.43]	[−.01, .26]
8. NTB	−.02 (−.03)	.00 (.00)	−.06 (.07)	**.21 (.25)**	**.33 (.37)**	.03 (.04)	**.37 (.43)**	–	[.07, .34]	[−.30, −.02]	[.04, .30]
9. Social Anx.	−.13 (−.13)	−.13 (−.16)	−.01 (−.01)	**−.65 (−.70)**	**.64 (−.70)**	**−.52 (−.60)**	**−.52 (−.58)**	**.21 (.23)**	–	[.31, .55]	[−.22, .05]
10. AQ Social Diff.	−.10 (−.11)	−.10 (−.13)	−.02 (−.02)	**.47 (−.56)**	**−.56 (−.66)**	**−.27 (−.34)**	**−.54 (−.66)**	**−.16 (−.21)**	**.44 (.50)**	–	[−.23, .06]
11. Sex	−.02 (−.01)	.04 (.05)	**−.17 (−.18)**	.12 (.11)	**.21 (.22)**	−.04 (−.04)	.13 (.13)	**.17 (.17)**	−.09 (−.10)	−.09 (−.09)	–

*Note.* Correlations in bold were statistically significant (*p* < .05). AQ = Autism Quotient, *N* = 181; All other variables, *N* = 203; CFMT% = Cambridge Face Memory Test % correct; CCMT% = Cambridge Car Memory Test % correct; Face Selective = CFMT controlling for CCMT. All variables are residuals, controlling for age. Reliability for residuals: CFMT% *α* = 0.86; Face-selective *α* = 0.73; CCMT% *α* = 0.86; Total Extraversion inter-item *a* = 0.92; Affiliative Extraversion *α* = 0.91; Agentic Extraversion α =0.81; Sociability *α* = 0.88; Need to Belong (NTB) *α* = 0.88; Social Anxiety α = 0.93; AQ-social difficulties *α* = 0.79; Internal consistency reliability for sex was set to 1 (i.e., perfect reliability). We had no a priori prediction regarding sex, but report here for completeness.

**Table 8:** Study 4: Descriptive statistics of measures for face recognition ability and personality traits (N = 2028).

Measures	Min	Max	Mean	*SD*	Skew	Kurtosis	α

CFMT%	33.33	100.00	75.49	13.54	−0.308	−0.643	0.90
BFI – Extraversion	0	32	15.45	6.19	0.148	−0.445	0.85
BFI – Openness	9	41	28.06	4.94	−0.267	0.095	0.69
BFI – Conscientiousness	2	36	20.19	6.08	−0.005	−0.303	0.83
BFI – Agreeableness	2	36	22.54	5.23	−0.245	0.093	0.74
BFI – Neuroticism	0	32	14.54	5.94	0.089	−0.271	0.83

Note. BFI = Big Five Inventory.

**Table 9:** Study 4: Intercorrelations (Corrected Correlations in Parentheses) Among Measures of Face Recognition Ability and Personality Traits, Age Controlled for, with 95% Confidence Intervals Above the Diagonal (N = 2028).

Measures	1.	2.	3.	4.	5.	6.	7.

1. CFMT%	–	[−.00, .08]	[.03, .11]	[−.04, .04]	[−.06, .02]	[−.02, .06]	[−.00, .08]
2. BFI –Extraversion	.04 (.05)	–	[.12, .20]	[.15, .23]	[.19, .27]	[.24, .32]	[−.04, .04]
3. BFI –Openness	**.07 (.09)**	**.16 (.21)**	–	[−.00, .08]	[.06, .14]	[.04, .12]	[−.06, .02]
4. BFI –Conscientiousness	.00 (.00)	**.19 (.24)**	.04 (.06)	–	[.23, .31]	[.25, .33]	[−.01, .07]
5. BFI –Agreeableness	−.02 (−.03)	**.23 (.30)**	**.10 (.15)**	**.27 (.37)**	–	[.21, .29]	[.01, .09]
6. BFI –Neuroticism	.02 (.02)	**.28 (.34)**	**.08 (.14)**	**.29 (.37)**	**.25 (.33)**	–	[−.22, −.14]
7. Sex	.04 (.04)	.00 (.00)	−.02 (−.02)	.03 (.03)	**.05 (.06)**	**−.18, (−.20)**	–

*Note.* Correlations in bold were statistically significant (*p* < .05). All variables are residuals, after controlling for age. Reliability estimates for residuals: CFMT% *α* = 0.86; Extraversion *α* = 0.84; Openness *α* = 0.67; Conscientiousness *α* = 0.77; Agreeableness *α* = 0.70 and Neuroticism *α* = 0.80. Internal consistency reliability cannot be calculated for sex so was set to 1 (perfect reliability). We had no a priori predictions regarding sex but report its correlation with all variables in the study for completeness.

## Data Availability

Raw data for all studies is publicly available on the Open Science Framework (OSF) repository at https://osf.io/4bx7d/.

## Footnotes

1 Data for Study 1 and 2 were collected in 2014–2015, when Facebook was the most widely used social networking platform.

2 The meta-analysis was conducted using the ‘metafor’ package (version 4.2–0) (Viechtbauer, 2010) in the R statistical software (version 2022–09-08). The sample-size-weighted mean correlation and corresponding 95% confidence interval (CI) were estimated using the inverse variance method based on the random-effects model. The heterogeneity among the studies was assessed using the Q statistic and the I^2 statistic.

3 The participant information collected in these studies used the question ‘Sex (please circle)’ with response options ‘Female’ and ‘Male.’ We are aware that this binary format does not fully encompass the range of gender identities. In recent years, inclusive options that allow participants to self-identify their gender have become more widely recognised for promoting inclusivity in research practices.

4 We controlled for the influence of age due to previously reported different age curves for face recognition ability (Germine et al., 2011). To do this we regressed age out of all variables by calculating residual values from a first-order regression.

5 Sex was significantly, positively associated with both FF performance and NSSQ-N in favour of females. However, a partial correlation controlling for sex remained significant, indicating sex was not driving the association between FF performance and the NSSQ-N.

6 Sex was significantly, positively associated with both familiar face recognition performance and NSSQ-N in favour of females. However, a partial correlation controlling for sex remained significant, indicating sex was not driving the association between familiar face recognition performance and the NSSQ-N.

7 This inconsistency may be due to the methodologies employed to estimate online SNS. It is possible that the estimate of Facebook friends was more accurate in Study 2, as participants were consistently asked to verify their Facebook account, unlike in Study 1 where we relied on participants’ self-report. This is, however, speculative and warrants further investigation.
