# Supplementary material for: Face recognition’s practical relevance: Social bonds, not social butterflies
Source: Cognition. Author manuscript; Available in PMC 2024 Oct 2. (PMC11445692; doi:10.1016/j.cognition.2024.105816)
Supplement: Supplement - Appendix A [file NIHMS2022661-supplement-Supplement_-_Appendix_A.docx]

**Supplementary Materials**

***Table S1.*** *Study 1: Bayesian Factors (BF_01_) for Correlations Among Measures of Face Recognition Ability and Social Network Size, with 95% Credible Intervals Above the Diagonal (N = 93)*

|  | CFMT% | FF% | SNI | Dunbar-N | FB | NSSQ-N | Sex |
| --- | --- | --- | --- | --- | --- | --- | --- |
| 1. CFMT% | ̶ | [0.46-.0.72] | [-.18-.22] | [-.20-.21] | [-.26-.14] | [.02-.40] | [-.12-.27] |
| 2. FF% | 0.00 |  | [-.14-.26] | [-.22-.18] | [-.23-.17] | [.10-.47] | [.08-.45] |
| 3. SNI | 7.54 | 6.61 | ̶ | [-.24-.16] | [-.03-.36] | [.15-51] | [-.20-.21] |
| 4. Dunbar-N | 7.71 | 7.57 | 7.04 | ̶ | [-.06-.33] | [-.16-.24] | [-.21-.20] |
| 5. FB | 6.42 | 7.29 | 2.09 | 3.01 | ̶ | [-.11-.29] | [-.20-20] |
| 6. NSSQ-N | 0.84 | 0.12 | 0.02 | 7.05 | 5.06 | ̶ | [.19-54] |
| 7. Sex | 5.83 | 0.20 | 7.71 | 7.71 | 7.72 | 0.01 | ̶ |

*Note.* Bayes Factors reported are BF01, where values < 1 indicate evidence for the alternative hypothesis and values > 1 indicate evidence for the null.

***Table S2.***

*Study 2: Bayesian Factors (BF_01_) for Correlations Among Measures of Face Recognition Ability, Social Network Size and Extraversion, with 95% Credible Intervals Above the Diagonal (N =101)*

| Measures | CFMT | FF | SNI | FB | NSSQ | Extra | Affiliative | Agentic | Sex |
| --- | --- | --- | --- | --- | --- | --- | --- | --- | --- |
| 1. CFMT% | ̶ | [.25-.57] | [-.26-.13] | [-.11-.28] | [.03-.40] | [-.21-.18] | [-.16-.22] | [-.25-.14] | [-.17-.22] |
| 2. FF% | 0.00 | - | [-.00-.37] | [.15-50] | [.04-.41] | [.03-.40] | [.04-.40] | [-.05-.33] | [.03-.40] |
| 3. SNI | 6.35 | 1.14 | ̶ | [.14-.48] | [.06-.43] | [.12-.48] | [.12-.47] | [.03-.40] | [-.17-.22] |
| 4. FB | 5.49 | 0.02 | 0.03 | ̶ | [-.08-.31] | [.12-.48] | [.06-.43] | [.08-.45] | [-.22-.17] |
| 5. NSSQ-N | 0.63 | 0.51 | 0.28 | 4.00 | ̶ | [-.06-.32] | [-.07-.31] | [-.09-.29] | [-.02-.35] |
| 6. Extraversion | 7.96 | 0.66 | 0.05 | 0.08 | 3.29 | ̶ | [.80-.91] | [.78-.89] | [-.12-.27] |
| 7. Affiliative | 7.67 | 0.55 | 0.06 | 0.27 | 3.81 | 0.00 | ̶ | [.29-.60] | [-.03-.34] |
| 8. Agentic | 6.82 | 2.70 | 0.61 | 0.28 | 4.58 | 0.00 | 0.00 | ̶ | [-.22-.16] |
| 9. Sex | 7.73 | 0.60 | 7.73 | 8.03 | 1.83 | 5.90 | 2.21 | 7.67 | ̶ |

*Note.* Bayes Factors reported are BF01, where values < 1 indicate evidence for the alternative hypothesis and values > 1 indicate evidence for the null.

***Table S3.***

*Study 3: Bayesian Factors (BF_01_) for Correlations Among Measures of Face Recognition Ability and Personality Traits, with 95% Credible Intervals Above the Diagonal (N = 203)*

| Measures | 1 | 2 | 3 | 4 | 5 | 6 | 7 | 8 | 9 | 10 |
| --- | --- | --- | --- | --- | --- | --- | --- | --- | --- | --- |
| 1. CFMT% | ̶ | [.18, .43] | [-.11, .17] | [-.10, .18] | [-.12, .16] | [-.07, .21] | [-.16, .11] | [-.26, .01] | [-.23 .06] | [-.16, .12] |
| 2. CCMT% | 0.00 | ̶ | [-.14, .13] | [-.18, .09] | [-.08, .19] | [-.15, .12] | [-.20, .07] | [-.15, .13] | [-.16, .13] | [-.30, -.04] |
| 3. Tot. Ext. | 10.26 | 11.38 | ̶ | [.90, .94] | [.83, .90] | [.67, .80] | [.08, .34] | [-.72, -.56] | [-.58, -.35] | [-.02, .25] |
| 4. Affil. Ext. | 9.59 | 9.10 | 0.00 | ̶ | [.51, .69] | [.76, .85] | [.20, .44] | [-.71, -.54] | [-.65, -.45] | [.08, .34] |
| 5. Agent. Ext. | 11.01 | 7.99 | 0.00 | 0.00 | ̶ | [.38, .59] | [-.11, .16] | [-.61, -.41] | [-.40, -.13] | [-.17, .10] |
| 6. Sociability | 6.91 | 11.20 | 0.00 | 0.00 | 0.00 | ̶ | [.25, .49] | [-.61, -.40] | [-.64, -.43] | [.00, .27] |
| 7. NTB | 10.76 | 7.52 | 0.11 | 1.41e0-4 | 10.75 | 2.98e0-6 | ̶ | [.08, .34] | [-.31, -.03] | [.04, .31] |
| 8. Social Anx. | 2.06 | 11.23 | 0.00 | 0.00 | 0.00 | 0.00 | 0.10 | ̶ | [.30, .54] | [-.22, .05] |
| 9. AQ S. Diff. | 5.32 | 10.38 | 1.70e0-9 | 0.00 | 0.01 | 0.00 | 0.73 | 0.00 | ̶ | [-.24, .05] |
| 10. Sex | 10.95 | 0.51 | 2.80 | 0.09 | 9.90 | 1.89 | 0.46 | 5.51 | 4.55 | ̶ |

*Note.* Bayes Factors reported are BF01, where values < 1 indicate evidence for the alternative hypothesis and values > 1 indicate evidence for the null.

***Table S4.***

*Study 4: Bayesian Factors (BF_01_) for Correlations Among Measures of Face Recognition Ability and Personality Traits, with 95% Credible Intervals Above the Diagonal (N = 2,028)*

| Measures | 1. | 2. | 3. | 4. | 5. | 6. | 7. |
| --- | --- | --- | --- | --- | --- | --- | --- |
| 1. CFMT% | - | [-.02, .07] | [.05, .13] | [-.04, .05] | [-.05, .04] | [-.03, .06] | [-.04, .04] |
| 2. BFI –Extraversion | 21.11 | - | [.12, .20] | [.16, .24] | [.19, .27] | [.25, .33] | [-.05, .04] |
| 3. BFI –Openness | 0.01 | 0.00 | - | [.01, .10] | [.06, .15] | [.04, .13] | [-.07, .02] |
| 4. BFI –Conscientiousness | 33.54 | 0.00 | 2.13 | - | [.22, .31] | [.27, .35] | [-.04, .04] |
| 5. BFI –Agreeableness | 34.69 | 0.00 | 0.00 | 0.00 | - | [.22, .31] | [-.01, .08] |
| 6. BFI –Neuroticism | 25.50 | 0.00 | 0.02 | 0.00 | 0.00 | - | [-.24, -.15] |
| 7. Sex | 35.90 | 35.50 | 18.16 | 35.90 | 10.70 | 0.00 | - |

*Note.* Bayes Factors reported are BF01, where values < 1 indicate evidence for the alternative hypothesis and values > 1 indicate evidence for the null.

**References**

**[1]** The jamovi project (2021). *jamovi*. (Version 2.2) [Computer Software]. Retrieved from <https://www.jamovi.org>.

**[2]** R Core Team (2021). *R: A Language and environment for statistical computing*. (Version 4.0) [Computer software]. Retrieved from <https://cran.r-project.org>. (R packages retrieved from MRAN snapshot 2021-04-01).

**[3]** JASP Team (2018). *JASP*. [Computer software]. Retrieved from <https://jasp-stats.org>.

**[4]** Ly, A., Verhagen, A. J. & Wagenmakers, E.-J. (2016). Harold Jeffreys. *Journal of Mathematical Psychology, 72*, 19-32.

**[5]** Ly, A., Marsman, M., Wagenmakers, E.-J. (2018). Analytic Posteriors for Pearson’s Correlation Coefficient. *Statistica Neerlandica, 72*(1), 4-13.

***Table S******5***

*Study 1. Intercorrelations (Corrected Correlations in Parentheses) Among Measures of Face Recognition Ability, Expression Labelling Ability and Social Network Size, Age Controlled for, with 95% Confidence Intervals Above the Diagonal (N = 93)*

|  | CFMT% | FF% | Expression | SNI | Dunbar-N | FB | NSSQ-N | Sex |
| --- | --- | --- | --- | --- | --- | --- | --- | --- |
| CFMT% | ̶ | [.44-.73] | [.26, .59] | [-.18-.23] | [-.20-.21] | [-.26-.15] | [.012-.40] | [-.13-.28] |
| FF% | **.62 (.69)** | ̶ | [.17, .53] | [-.14-.27] | [-.21-.20] | [-.21-.19] | [.09-.46] | [.08-.46] |
| Expression% | **.44 (.52**) | **.36 (.43)** | ̶ | [-.09, .31] | [-.08, .33] | [-.19, .22] | [-.22, .19] | [.10, .47] |
| SNI | .03 (.03) | .07 (.07) | .12 (.14) | ̶ | [-.25-.16] | [-.04-.36] | [.16-52] | [-.20-.21] |
| Dunbar-N | .009 (.01) | -.01 (-.01) | .13 (.15) | -.05 | ̶ | [-.08-.32] | [-.15-.25] | [-.22-.19] |
| FB | -.06 (-.06) | -.01 (-.01) | .01 (.01) | .17 | .13 | ̶ | [-.08-.32] | [-.22-19] |
| NSSQ-N | **.22 (.23)** | **.29 (.31)** | -.01 (-.01) | **.36** | .05 | .10 | ̶ | [.21-55] |
| Sex | .08 (.08) | **.28 (.30)** | **.30 (.34)** | .00^a^ | -.01 | .01^a^ | **.39** | ̶ |

Note. CFMT% = Cambridge Face Memory Test % correct; FF% = Famous Faces Test % correct of those known; Expression% = Expression Labelling task % correct; SNI = Social Network Index; FB = Facebook friends; NSSQ-N = Norbeck Social Support Questionnaire. Correlations in bold were statistically significant (p > .05). All variables are residuals, after controlling for age. Reliability for residuals: CFMT% α = .89; FF% α = .90; Expression Labelling α = .79; Extraversion α =.84; Affiliative Extraversion, α = .85, Agentic Extraversion, α =.69.  ^a^Internal consistency reliability could not be calculated for other variables so was set to 1 (perfect reliability). We had no a priori prediction regarding sex, but report here for completeness.

***Table S6***

*Study 3. Intercorrelations (Corrected Correlations in Parentheses) Among Measures of Face Recognition Ability, Expression Labelling Ability and Personality Traits, Age Controlled for, with 95% Confidence Intervals Above the Diagonal (N = 203)*

| Measures | 1 | 2 | 3 | 4 | 5 | 6 | 7 | 8 | 9 | 10 | 11 | 12 |
| --- | --- | --- | --- | --- | --- | --- | --- | --- | --- | --- | --- | --- |
| 1. CFMT% | ̶ | [.93, .96] | [.18, .43] | [.03, .30] | [.11, .40] | [.07, .37] | [-.11, .17] | [-.07, .21] | [-.17, .11] | [-.26, .01] | [-.22 .05] | [-.15, .13] |
| 2. Face Sel. | **.95 (1.00)** | ̶ | [-.14, .14] | [-.01, .27] | [.12, .41] | [.07, .37] | [-.10, .18] | [-.05, .22] | [-.14, .14] | [-26, .01] | [-.23 .04] | [-.10, .18] |
| 3. CCMT% | **.31 (.36)** | .00 (.05) | ̶ | [-.03, .25] | [-.16, .16] | [-.13, .18] | [-.14, .14] | [-.15, .13] | [-.20, .08] | [-.15, .13] | [-.16, .12] | [-.30, -.03] |
| 4. Expression % | **.17 (.21)** | .13 (.17) | .11 (.13) | ̶ | [-.24, .29] | [-.42, .09] | [-.10, .18] | [-.18, .10] | [-.09, .19] | [-.24, .03] | [-.16, .14] | [.07, .34] |
| 5. Face Identity Aftereffects | **.26 (.39)** | **.27 (.44)** | .00 (.00) | .03 (.05) | ̶ | [.02, .32] | [-.21, .10] | [-.32, -.01] | [-.25, .07] | [-.21, .11] | [-.03, .30] | [-.14, .18] |
| 6. Composite Face Effect | **.23 (.30)** | **.23 (.33)** | .03 (.04) | **.18 (.25)** | **.18 (.30)** | ̶ | [-.30, .01] | [-.18, .14] | [-.21, .10] | [-.10, .22] | [.01, .33] | [-.25, .06] |
| 7. Tot. Ext. | .03 (.03) | .04 (.00) | -.00 (-.00) | .04 (.05) | -.06 (-.09) | -.14 (-.18) | ̶ | [.68, .80] | [.09, .35] | [-.72, -.56] | [-.58, -.37] | [-.03, .24] |
| 8. Sociability | .08 (.08) | .09 (.11) | -.01 (-.01) | -.04 (-.05) | **-.17 (-.25)** | -.02 (-.03) | **.75 (.84)** | ̶ | [.25, .48] | [-.61, -.41] | [-.63, -.43] | [-.02, .25] |
| 9. NTB | -.02 (-.03) | .00 (.00) | -.06 (.07) | .05 (.06) | -.09 (-.13) | -.06 (-.08) | **.21 (.25)** | **.37 (.43)** | ̶ | [.07, .34] | [-.30, -.03] | [.02, .29] |
| 10. Social Anx. | -.13 (-.13) | -.13 (-.16) | -.01 (-.01) | -.11 (.13) | -.05 (-.07) | .06 (.08) | **-.65 (-.70)** | **-.52 (-.58)** | **.21 (.23)** | ̶ | [.31, .54] | [.22, .05] |
| 11. AQ S. Diff. | -.10 (-.11) | -.10 (-.13) | -.02 (-.02) | -.01 (-.01) | .14 (.22) | **.17 (.23)** | **-.47 (-.56)** | **-.54 (-.66)** | **-.16 (-.21)** | **.44 (.50)** | ̶ | [.22, .06] |
| 12. Sex | -.02 (-.01) | .04 (.05) | **-.17 (-.18)** | **.21 (.24)** | .02 (.03) | -.10 (-.12) | .12 (.11) | .13 (.13) | **.17 (.17)** | -.09 (-.10) | -.09 (-.09) | ̶ |

*Note*. Correlations in bold were statistically significant (*p* < .05). AQ = Autism Quotient, *N* = 181; Face Identity Aftereffects and Composite Effect N = 158; All other variables *N* = 203; CFMT% = Cambridge Face Memory Test % correct; Face Selective = CFMT controlling for CCMT; CCMT% = Cambridge Car Memory Test % correct; Expression% = Expression Labelling task % correct. All variables are residuals, controlling for age. Reliability estimates: CFMT% *α* = .86; Face-selective *α* = .73; CCMT% *α* = .86; Expression Labelling α = .79; Face Identity Aftereffects α = .52; Composite Face Effect α = .67; Total Extraversion inter-item *α* =.92; Sociability *α* =.88; Need to Belong (NTB) *α* = .88; Social Anxiety α = .93; AQ-social difficulties *α* = .79; Internal consistency reliability for sex was set to 1 (i.e., perfect reliability). We had no a priori prediction regarding sex, but report here for completeness.
